# Yeast as a Model to Unravel New BRCA2 Functions in Cell Metabolism

**DOI:** 10.3389/fonc.2022.908442

**Published:** 2022-06-06

**Authors:** Alessandra Costanza, Nicoletta Guaragnella, Antonella Bobba, Caterina Manzari, Alberto L’Abbate, Claudio Lo Giudice, Ernesto Picardi, Anna Maria D’Erchia, Graziano Pesole, Sergio Giannattasio

**Affiliations:** ^1^ Institute of Biomembranes, Bioenergetics and Molecular Biotechnologies, National Research Council, Bari, Italy; ^2^ Department of Biosciences, Biotechnologies and Biopharmaceutics, University of Bari “Aldo Moro”, Bari, Italy

**Keywords:** BRCA2, metabolic reprogramming, yeast, RNA seq, mitochondrial dysfunction, mitochondrial retrograde pathway

## Abstract

Mutations in BRCA2 gene increase the risk for breast cancer and for other cancer types, including pancreatic and prostate cancer. Since its first identification as an oncosupressor in 1995, the best-characterized function of BRCA2 is in the repair of DNA double-strand breaks (DSBs) by homologous recombination. BRCA2 directly interacts with both RAD51 and single-stranded DNA, mediating loading of RAD51 recombinase to sites of single-stranded DNA. In the absence of an efficient homologous recombination pathway, DSBs accumulate resulting in genome instability, thus supporting tumorigenesis. Yet the precise mechanism by which BRCA2 exerts its tumor suppressor function remains unclear. BRCA2 has also been involved in other biological functions including protection of telomere integrity and stalled replication forks, cell cycle progression, transcriptional control and mitophagy. Recently, we and others have reported a role of BRCA2 in modulating cell death programs through a molecular mechanism conserved in yeast and mammals. Here we hypothesize that BRCA2 is a multifunctional protein which exerts specific functions depending on cell stress response pathway. Based on a differential RNA sequencing analysis carried out on yeast cells either growing or undergoing a regulated cell death process, either in the absence or in the presence of BRCA2, we suggest that BRCA2 causes central carbon metabolism reprogramming in response to death stimuli and encourage further investigation on the role of metabolic reprogramming in BRCA2 oncosuppressive function.

## Introduction

*BRCA2* (*BReast CAncer susceptibility gene 2*) was identified in 1995 (1) based on its most striking clinical manifestation: mutations in this gene increase the risk for breast cancer. Approximately 5–10% of individuals that develop breast cancer are genetically predisposed to the disease, and, among these, 10–30% have been attributed to mutations in the tumor suppressor gene *BRCA2*. The penetrance of having *BRCA2* mutations is severe – there is a 45-69% risk of developing breast cancer by 70-80 years of age for women carrying a pathogenic BRCA2 variant against a 13% risk for women in the general population (https://www.cancer.gov/about-cancer/causes-prevention/genetics/brca-fact-sheet#r4) (2). In addition, women inheriting pathogenic *BRCA2* mutation/s have a considerably increased risk to develop ovarian cancer (11-17% risk *versus* 1.2% in the general population) (https://www.cancer.gov/about-cancer/causes-prevention/genetics/brca-fact-sheet#r4) (2). *BRCA2* mutations confer an increased risk for several other cancer types, including pancreatic (3) and prostate cancer (4).

The expression of BRCA2 is tightly regulated during cell cycle progression: BRCA2 steady state levels are low in resting cells and G1-phase cycling cells and increase in S-phase (5). In 1997, yeast two-hybrid screenings demonstrated the interaction of BRCA2 with RAD51 (6), a recombinase essential in the mechanism of homologous recombination (HR). BRCA2 involvement in HR began to be hypothesized.

*BRCA2* is localized on the long arm of chromosome 13 (13q12.3) and is composed of 27 exons, which encode a multi-domain protein of 3,418 amino acids. Structural/functional BRCA2 domains have been identified: the repeated BRC domains, which consist of 8 repeated conserved motifs of about 35 amino acids each that interact with monomeric RAD51; an N-terminal region that mediates protein-protein interactions, including binding to PALB2 and EMSY; a DNA-binding domain (DBD) that contains a helical domain (H), three oligonucleotide binding (OB) folds and a tower domain (T), which may facilitate BRCA2 binding to both single-stranded DNA (ssDNA) and double-stranded DNA (dsDNA). This region also associates with deleted in split-hand/split-foot syndrome (DSS1), a protein that stabilizes BRCA2. A phenylalanine-proline-proline motif adjacent to the DBD binds FANCD2 and DMC1. The C-terminus contains two nuclear localization signals (NLSs) and the TR2 domain, which interacts with RAD51 nucleofilaments stabilizing RAD51 oligomerization (for a comprehensive review on BRCA2 structure see (7)). BRCA2 is primarily a nuclear protein, and its subcellular localization is controlled by two NLSs and one nuclear export signal (NES). BRCA2 can recruit numerous regulatory proteins including RAD51, PALB2, BCCIP, EMSY and FANCD2 (8, 9); this supports the hypothesis that BRCA2 is a multifunctional protein involved in numerous cellular pathways. In this article, we will briefly go through the role of BRCA2 in HR and present preliminary data obtained by differential RNA-seq analysis supporting the existence of new roles of BRCA2 in cell death regulation and metabolic reprogramming opening new avenues of investigations on the oncosuppressive role of BRCA2.

## Biological Functions of Brca2: Studies in Mammalian Cells

### 1.a BRCA2 in HR

The first and best-characterized function of BRCA2 is in the repair of DNA double-strand breaks (DSBs) by HR. Several excellent reviews have described in detail the role of BRCA2 in HR (7, 10–13). Here, we will only briefly summarize it. DSBs occur during DNA replication or during exposure to genotoxic drugs and ionizing radiation. Mammalian cells repair DSBs by HR, an error-free repair pathway, or by non-homologous end-joining (NHEJ), an error-prone repair pathway. In HR, DSBs are first resected by the MRN complex (Mre11-RAD50-Nbs1) and its interacting partner CtIP, a protein with nuclease activity. The resection generates a single-stranded DNA tail that is protected from degradation by replication protein A (RPA). BRCA2 recruits the recombinase RAD51 to DSBs. Briefly, BRCA2 directly interacts with both RAD51 and single-stranded DNA, mediating loading of RAD51 recombinase to sites of single-stranded DNA. Here, RAD51 displaces RPA and catalyzes homologous pairing and DNA strand exchange. In the absence of an efficient HR pathway, DSBs accumulate resulting in genome instability, thus supporting tumorigenesis (14).

Besides mitosis, HR is also required during meiosis. Meiotic DSBs are formed by a nuclease complex containing SPO11 and TOPOVIBL (15). Their repair requires HR, which occurs essentially as for HR of somatic cells. However, despite sharing many HR components with somatic cells, meiotic cells have adapted specific steps to avoid use of the sister chromatid as the repair template. This meiotic adaptation of HR requires specialized meiotic protein paralogs, such as DMC1, a meiosis-specific paralog of RAD51 (16). Brandsma et al. (17) recently described a highly evolutionarily conserved interaction of BRCA2 with HSF2BP, a protein originally identified only in testis. HSF2BP is transcribed in human cancer cell lines and overexpressed in some tumor samples. Intriguingly, loss of HSF2BP in mice disrupts meiotic HR during spermatogenesis resulting in male infertility. HSF2BP-BRCA2 interaction occurs within Gly2270 and Thr2337, a region between the BRC repeats and the DNA binding domain of BRCA2 that does not harbor known cancer-associated mutations, suggesting that this region may have a specific function in reproduction rather than in cancer pathogenesis.

### 1.b BRCA2 Protects Telomere Integrity and Stalled Replication Forks

Telomeres consist of repetitive G-rich nucleotide sequences and associated proteins at the ends of linear chromosomes. The main function of telomeres is to protect chromosome ends from degradation and chromosomal end-to-end fusions, breaks and rearrangements. BRCA2 localizes to telomeres in S phase and loads RAD51 on telomeres during S/G2 phase protecting telomere integrity. Accumulation of telomere dysfunction and shortening is a peculiarity of *BRCA2*-deficient cells (18, 19). This function is directly linked to the BRCA2 role in global replication fork protection. Telomeres are composed of TTAGGG repeats, which can form secondary structures, such as G-quadruplex, which make telomere lagging strands susceptible to fork stalling. Besides secondary structures (G-quadruplex and R-loops), progression of the replication forks can be inhibited by several factors, including DNA lesions, repetitive sequences, and imbalance of the nucleotides’ pool (20–23). BRCA2 prevents degradation of nascent DNA at stalled replication forks by loading RAD51 onto damaged DNA (24, 25), thus promoting resolution of fork stalling and supporting genome stability.

### 1.c BRCA2 in Cell Cycle Progression

A growing number of evidence suggests that BRCA2 may regulate cell cycle progression. In 2001, Marmostein et al. (26) demonstrated that a protein complex containing BRCA2 and BRAF35 associates with condensed chromatin concomitantly with histone H3 phosphorylation, *i.e.* during mitosis. Interestingly, microinjection of anti-BRAF35 or anti-BRCA2 antibodies into synchronized HeLa cancer cells delayed entry in mitosis, suggesting a role for BRCA2 in cell cycle progression. Subsequent studies showed that BRCA2 binds to BubR1, a mitotic checkpoint kinase that ensures equal chromosome segregation in mitosis (27). BRCA2 functions as a scaffold protein that recruits PCAF acetyltransferase on the kinetochore. In turn, PCAF acetylates BubR1 activating it as a key regulator of spindle assembly checkpoint. In mice, prevention of BRCA2-BubR1 interaction by ectopic expression of the BRCA2 domain that binds BubR1, resulted in spontaneous tumorigenesis without affecting HR (27), supporting a direct role of BRCA2 in mitosis. Additional evidence demonstrated that completion of cytokinesis requires BRCA2: BRCA2 is recruited to the midbody by interacting with Filamin A, an actin-binding protein. At the midbody, BRCA2 participates to recruitment of endosomal sorting complex and formation of CEP55-containing complexes necessary for abscission. Mutations in BRCA2 that disrupt these interactions result in cytokinetic defects without affecting DNA repair by HR (28). Taken together, these findings highlight a role for BRCA2 in regulating proper cell division, separately from DNA damage repair.

### 1.d BRCA2 and Transcriptional Control

There is evidence that BRCA2, besides being involved in DNA repair and cell division, may control transcription. Shin et al. (29) have shown that BRCA2 interacts with the nuclear receptor coactivator GRIP1 to promote transcription activation by androgen receptor (AR). In addition, BRCA2 cooperates with P/CAF and BRCA1 in a GRIP1-dependent manner to support AR transactivation. Mormostein et al. (30) have reported that BRCA2 interacts with p53 in cancer cells inhibiting p53 transcriptional activity, thus supporting another mechanism of BRCA2-mediated tumor suppression. In addition to p53, BRCA2 associates to Smad3, an essential component in the signal transduction pathway mediated by TGFβ, and the complex synergizes in the regulation of transcription (31). Overall, this evidence indicates that BRCA2, forming complexes with specific proteins, may control transcription of genes implicated in tumor development and/or progression.

### 1.e BRCA2 in Mitophagy

An unexpected role of BRCA2 has been recently described in the context of mitophagy (32), a mitochondrial quality control mechanism that eliminates defective mitochondria by a specific autophagy process (33). si-RNA-mediated knockdown of BRCA2 or other proteins of the Fanconi Anemia pathway, inhibited clearance of mitochondria by Parkin-mediated mitophagy (32), suggesting an essential role of BRCA2 in mitochondria homeostasis (see also *YEAST AS A MODEL TO STUDY BRCA2 FUNCTIONS AND CELL DEATH PROGRAMS* below) and indicating that BRCA2 is essential for a cytoplasmic-localized function, i.e., mitophagy.

## Yeast as a Model to Study Brca2 Functions and Cell Death Programs

We and others have reported a potential role of BRCA2 in modulating cell death programs. Cheung et al. studied the effect of *BRCA2* deletion on induction of apoptosis in the T-cell lineage by generating mice with conditional repression of *Brca2* only in T cells (*tBrca2^-/-^* mice) (8). Brca2-deficient proliferating T cells exhibited an apoptotic response to DNA damage comparable to wild-type thymocytes, but they displayed an increased propensity to undergo spontaneous apoptosis, an event that involves p53 signaling. Similarly, Hay et al. generated mice with *Brca2* deletion in the cells of the adult small intestine and observed a p53-dependent increase in spontaneous apoptosis that lead to spontaneous depletion of *Brca2^-/-^* stem cells (34), suggesting a protective role of *Brca2* loss against tumorigenesis of the small intestine.

We have shown a new function of BRCA2 as a modulator of sensitivity to anoikis, a form of programmed cell death induced in epithelial cells upon detachment from the extracellular matrix (35). Suppression of BRCA2 expression by short hairpin RNA promoted resistance to anoikis in prostate, breast, and thyroid normal epithelial cells, accompanied by reduced caspase 3/7 levels and activity. Resistance to anoikis was associated with a delay in the initial burst of ROS levels. Treatment with the antioxidants N-acetylcysteine and ascorbic acid reduced sensitivity to anoikis in human cells expressing BRCA2, suggesting that BRCA2 may modulate the cell redox state.

Yeast has been used as a powerful tool to discover individual gene functions as well as gene and protein interactions, and have contributed to the understanding of fundamental cellular processes such as eukaryotic cell cycle control, autophagy, mitochondrial function and biogenesis, genetic instability, metabolic regulation and cell death [for ref see (36)]. The ability to functionally express heterologous genes in yeast cells has allowed the development of new and elegant approaches leading to detailed structure-function analysis of numerous mammalian genes including oncogenes and tumor suppressors (37, 38).

Orthologues of BRCA2 have been found in a variety of taxa, from fungus to plant, invertebrates, and vertebrate. Surprisingly, the degree of evolutionary conservation is remarkably low (mouse-human 58%), particularly when compared with the other known tumor suppressors, i.e. p53 (mouse-human 78-98%). On the contrary, a greater degree of conservation is observed in some portions of the protein, particularly in the BRC motif, a sequence of 30-40 amino acids. This region, together with the C-terminal region of BRCA2, binds RAD51. The presence of these conserved motifs allowed the identification of BRCA2 in other eukaryotic organisms. The significant difference in size among BRCA2 in eukaryotic non-vertebrate organisms is due to the different number of BRC domains. All BRCA2 homologues have at least one BRC domain. Human BRCA2 has eight BRC motifs, six are present in chicken BRCA2, four in *Arabidopsis thaliana*, whereas only a single BRC motif appears to be present in the *C. elegans* and *U. maydis* BRCA2 homologues (Brh2). There are evidence that Brh2 interacts with DSS1, crucial for its activity in DNA repair (39, 40).

*Saccharomyces cerevisiae* lacks a homologue of BRCA2 and is evolutionary distant from the basidiomycete fungus *U. maydis*. However, *S. cerevisiae* has SEM1 gene, homolog of DSS1 that interacts with BRCA2 in HR (41, 42). The lack of BRCA2 homologue makes yeast an ideal model organism to study its function. Indeed, BRCA2 gene has been successfully expressed in yeast and the effect of expression has been used to study spontaneous HR and DNA-damage-induced nuclear foci. Spugnesi et al. have demonstrated that expression of neutral and pathogenic variants of BRCA2 differentially affect recombinational event in yeast. Specifically, the expression of neutral BRCA2 variants stimulated yeast recombination, while the expression of the pathogenic variants did not (43).

In this context, we have used yeast to investigate the role of BRCA2 in cell death processes. Indeed, exponentially growing *S. cerevisiae* cells undergo regulated cell death (RCD) when exposed to 80 mM acetic acid (AA) in glucose-containing medium and 120 mM AA in galactose-containing medium (44–46). AA-RCD has been deeply investigated in our and other laboratories and shows many features of mitochondrial mammalian apoptosis [for reviews see (45, 46)], thus providing a suitable model to investigate the mechanisms and regulation of mitochondrial apoptotic pathways.

We have found that heterologous expression of human BRCA2 in yeast does not induce cell death by itself, but it can promote AA-RCD. Induction of BRCA2 expression increased the number of cells positive to different apoptotic markers, including DNA fragmentation and phosphatidylserine externalization *en route* to yeast AA-RCD (35). This result together with resistance to anoikis found in epithelial cells in which BRCA2 has been silenced (see above) open new perspectives to investigate additional BRCA2 functions, such as those involved in RCD regulation. Thus, we used yeast as a model for functional genomics studies to identify new genes with a potential role in BRCA2-mediated control of cell death processes. To this aim, we performed an RNA-seq analysis in yeast either growing or undergoing AA-RCD in the presence or absence of BRCA2 expression. All experimental procedures are reported as **Supplementary Material**.

Four different samples were analysed by RNA-seq (**Table 1**):

**Table 1:** Yeast samples analyzed.

	Y-BRCA2 (*a*)	Y-pYES (*a*)	Y-BRCA2-AA (*b*)	Y-BRCA2-CTRL (*b*)
pYES2	–	+	–	–
pYES2-BRCA2	+	–	+	+
SC-Gal	+	+	–	–
SC-Gal pH=3.00	–	–	+	+
AA 300 mM	–	–	+	–

a, 16 h incubation; b, 90 min incubation. The experimental conditions used for cell growth and death are explained in detail in **Supplementary Material**.

**Figure 1:**
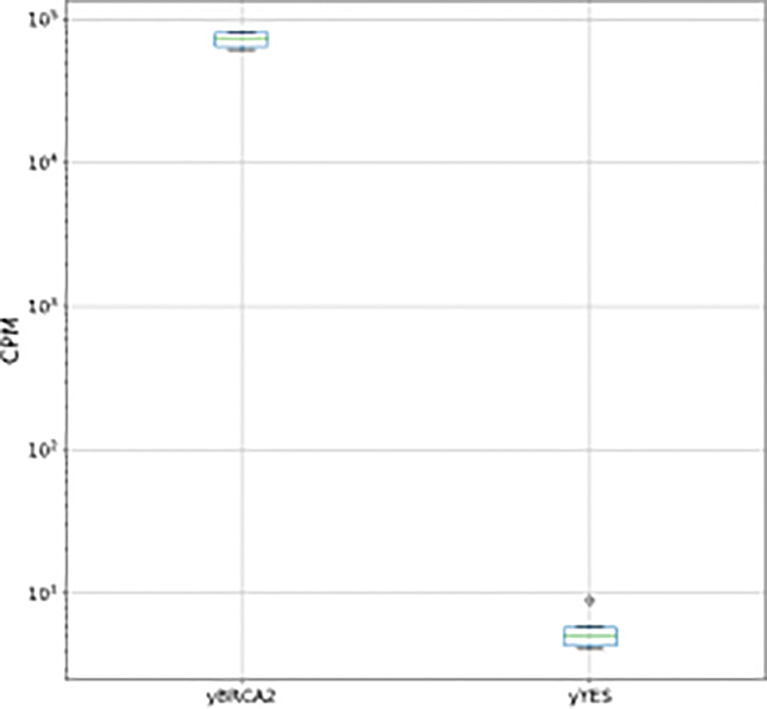
BCA2 normalized counts per million (CPM) for pairwise comparisons between Y- BRCA2 and Y-pYES.

1. Yeast cells harboring a recombinant pYES plasmid containing BRCA2 cDNA grown in SC-Gal for 16 hours **(Y-BRCA2)**;

2. Yeast cells harboring empty pYES plasmid grown in SC-Gal for 16 hours **(Y-pYES)**;

3. Yeast cells harboring a recombinant pYES plasmid containing BRCA2 cDNA treated with AA for 90 min **(Y-BRCA2-AA);**

4. Yeast cells harboring a recombinant pYES containing BRCA2 cDNA, cultured in the same medium without AA, as control **(Y-BRCA2-CTRL)**;

Firstly, we analyzed the effect of BRCA2 expression on yeast cells growing for 16 h up to stationary phase (comparison Y-BRCA2 vs Y-pYES). This analysis did not reveal any difference of gene expression between the two yeast cell types, but confirmed the up-regulation of BRCA2 (5.7 log2 fold change, Pval << 0.0001), ectopically expressed in Y-BRCA2 cells, as reported in **Figure 1**. This result was in good agreement with (35), showing that BRCA2 expression does not affect yeast cell growth, and as it was also indicated by the comparison of Y-BRCA2 and Y-pYES cell growth curves (data not shown).

**Figure 2:**
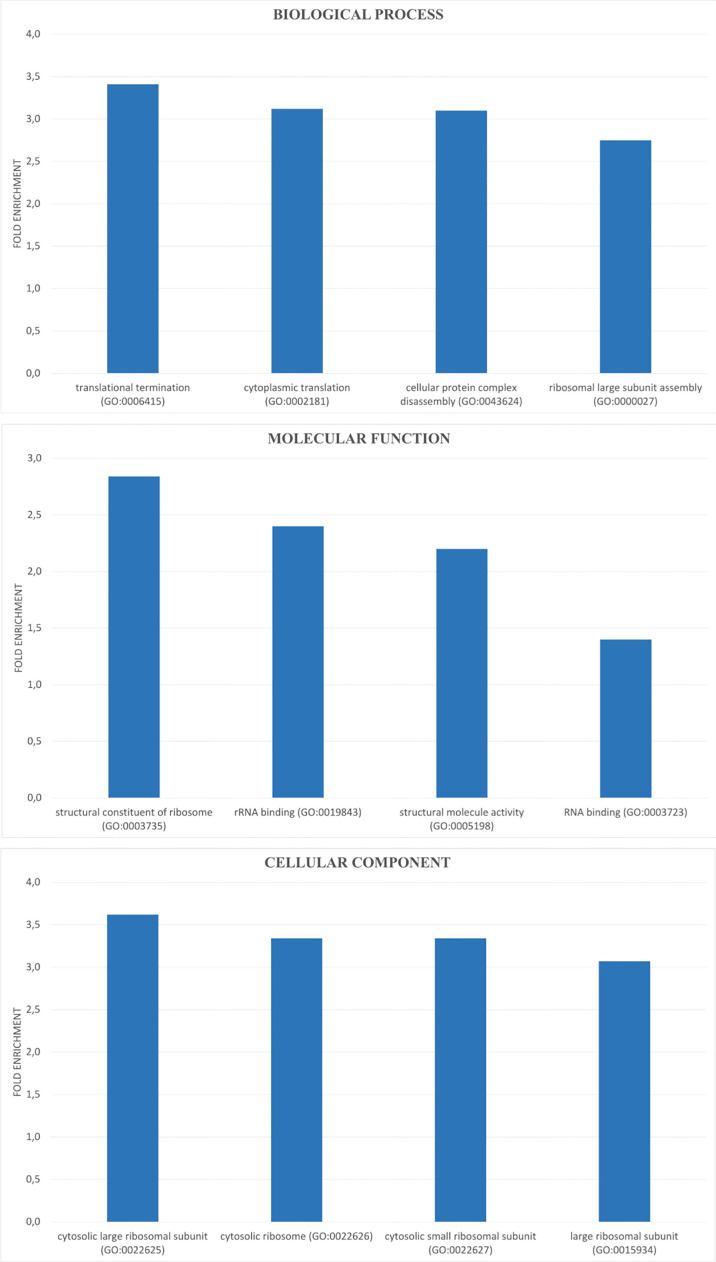
GO enrichment analysis of DEGs. DEGs were analyzed for enrichment in three GO ontologies (biological process, cellular component, and molecular function) and the top 4 over-represented for each category are shown as blue bar with the corresponding GO ID.

Then, we analyzed the effect of AA-RCD induction in yeast cells expressing BRCA2, by comparing Y-BRCA2-AA vs Y-BRCA2-CTRL cells. The differential expression analysis allowed us to identify 1724 deregulated genes (DEGs), whose expression resulted to be influenced by acetic acid treatment in yeast cells expressing BRCA2. Among them, 908 were upregulated and 816 downregulated. Of note, in this data set, identities and functions of 74% of DEGs are known (and only 26% are unknown). Statistically significant DEGs were used for a gene ontology (GO) search. A total number of 177 GO terms (FDR-adjusted p value<0.05) was enriched and distributed among the three GO categories as 120 biological process (BP), 7 molecular function (MF), and 50 cellular components (CC) (**Supplementary Table S1**). The top 4 over-represented BPs, MFs, and CCs are shown in **Figure 2**. Over-represented categories highlighted that most of the DEGs were involved in protein translation and ribosome biogenesis.

To understand the biological pathways more affected in Y-BRCA2 cells undergoing AA-RCD, we mapped all DEGs in the Kyoto Encyclopedia of Genes and Genomes (KEGG) database. **Supplementary Table S2** reports the list of DEGs for each KEGG pathway. The pathways with the higher numbers of DEGs are “metabolic pathways” with 202 genes, and “ribosome” with 137 genes. In the “metabolic pathways” we found up regulated genes involved in glutathione biosynthetic process and trehalose metabolic process. These genes are implicated in general response to stress (47–49). All genes in the “ribosome” pathway are down regulated. This is a common response to stress conditions in which proteins synthesis are strongly repressed. This aspect has previously been observed during different stress responses (47). Concomitantly, we found that several enzymes involved in biosynthesis and metabolism of purine and pyrimidine are down regulated. Consistently, in the “cell cycle” pathway 8 genes are up regulated, and 33 genes are down-regulated together with down-regulation of genes involved in DNA replication. Among the upregulated genes we found Cip1, which was identified as a negative regulator of cell cycle START and proposed to be an analog of human p21 (50, 51). With this respect, we interestingly found downregulation of Bub3, which is involved in the kinetochore checkpoint pathway through interaction with Mad2, Mad3 and Cdc20 (52). Mad3 is the yeast homolog of BubR1, which interacts with Bub3 and is acetylated in a BRCA2-dependent manner in the spindle assembly checkpoint (27).

The finding that cell cycle genes are repressed in yeast cells undergoing AA-RCD in the presence of BRCA2 is of particular interest when comparing it with a similar high-throughput study carried out in the same yeast strain undergoing AA-RCD without BRCA2 expression (53). In that case upon acetic acid stress, 29 up-regulated and 36 down-regulated genes involved in cell cycle were found with certain genes contributing to cell cycle progression while others likely causing the cell cycle arrest. Thus, in the absence of BRCA2 acetic acid caused a modulation, but not inhibition, of cell cycle whereas here we show that in the presence of BRCA2 acetic acid induce an apparent down-regulation of cell division genes. This suggests that in condition of acetic-acid stress BRCA2 promotes cell cycle arrest notwithstanding its role in progression and completion of cell division (see *BRCA2 in Cell Cycle Progression*).

Notwithstanding the key role of BRCA2 in HR, we also observed a general down regulation of genes involved in HR as well as mismatch and nucleotide excision repair pathways. With this respect, it is useful to compare our results with those obtained through differential RNA-seq-based transcriptomic and metabolomic analysis of the same yeast strain undergoing AA-RCD without BRCA2 expression (53). In this case 16 DEGs were found up regulated and only 4 downregulated in the “DNA repair” pathway, aiding to alleviate the cell death process. Thus, upon acetic acid stress, BRCA2 expression apparently did not stimulate DNA repair opposite to what happened in isogenic yeast cells without BRCA2 (53).

40 DEGs were identified in carbohydrate metabolism. Among the up-regulated genes there are the exose transporter *HTX1* and almost all glycolytic enzymes (*GLK1, HXK1, PGM2, FBA1, PGK1, GPM1, GPM2, TDH1, TDH2, TDH3, ENO1, ENO2, ERR2, ERR3, PYK2*). The already known inverse correlation between *HXK1* and *HXK2* expression, the latter known to localize to the nucleus causing repression of *HXK1* expression, is conserved in our experimental paradigm where HXK2 is downregulated. Although some enzymes of the pentose phosphate pathway (PPP), such as phosphoglucomutase (*PGM2*), 6-phosphogluconate dehydrogenase (*GND2*), 6-phosphogluconolactonase (*SOL4*) and transketolase (*TKL2*), are upregulated, glucose-6-phosphate dehydrogenase (*ZWF1*), the first regulatory enzyme of PPP, is not. This suggests that only the non-oxidative branch of PPP is activated but there is no shift of the glycolytic flux towards the PPP. In agreement with this, we found up-regulation of triose phosphate isomerase (*TPI1*) gene which is known to be a potent inhibitor of PPP. In fact, reduced activity of Tpi1 causes a reconfiguration of central metabolism, leading to increased flux of the PPP and increased oxidative stress resistance in yeast (54). This was shown to occur upon AA-RCD induction of the same yeast strain in the absence of BRCA2 as an extreme attempt to cope with cell death execution (55). Thus, BRCA2 expression apparently decreases yeast cell stress response to oxidative stress through *TPI1* up-regulation as already suggested in (35).

The significant up-regulation of the glycolytic pathway was accompanied by downregulation of genes encoding for tricarboxylic (TCA) cycle enzymes and their subunits (*ACO1*, *IDH1*, *IDH2*, *KGD2*, *SDH2* and *SDH3*). Interestingly, *CIT3*, coding for a methylcitrate synthase functional for propionate metabolism (56) was up-regulated. Propionate can exert its toxic function through methylcitrate which is a potent inhibitor of a variety of enzymes and cell growth in other organisms (57).

While the glycolytic enzymes are up-regulated in AA-RCD BRCA2-expressing cells, the expression of several OXPHOS enzymes and/or subunits are down-regulated: *SDH1*, *SDH2* and *SDH3*, ubiquinol cytochrome-c reductase complex (*QCR2*, *QCR6*, *QCR7*, *RIP1*, *COR1*), *CYT1*, *ATP3* and *ATP19*, *COX5A*, *COX6*, *COX12*. *CYC1* mRNA is strongly downregulated (-4.22 folds) as compared to the above-mentioned genes (ranging from -1.59 e -2.8 folds), suggesting that BRCA2 expression may promote mitochondrial dysfunction under stress conditions. Notably, only two subunits of cytochrome c oxidase (*COX1*, *COX5B*) and *CYC7*, the paralog of *CYC1*, are up regulated. It is puzzling that *HAP1* is up-regulated in AA-RCD BRCA2-expressing cells since *HAP1* has been shown to induce a metabolic switch from fermentation to respiration even under conditions of glucose repression due to increased heme synthesis (58).

Down-regulation of mitochondrial genes together with increase in glycolytic flux in yeast cells expressing BRCA2 after 90 min of acetic acid treatment differs from the metabolic state of the same cells without BRCA2 expression, in which both glycolysis and oxidative phosphorylation were found to be suppressed upon acetic acid treatment (53).

Concomitant with down-regulation of mitochondrial enzymes, we found up-regulation of *CAT2, YAT1, YAT2* involved in acetyl-CoA conversion to acetyl-carnitine as well as peroxisomal citrate synthase (*CIT2*). Up-regulation of *CIT2, DLD3* and *HOG1* together with pleiotropic drug resistance genes *PDR8*, *10*, *11*, *12* and *15* (59) indicates activation of mitochondrial retrograde (RTG) response following mitochondrial dysfunction (60, 61) leading to proliferation of peroxisomes with concomitant high expression of genes involved in fatty acid β-oxidation that can lead to increased acetyl-CoA production in the cytoplasm. Interestingly, *TUP1* and *CYC8*, which encode for a protein complex functional in another regulatory branch of *CIT2* transcription in response to glucose level [for refs see (61)], are both down-regulated.

## Discussion

Intensive investigations into the mechanisms through which BRCA2 together with BRCA1 work as cancer suppressors have shown their key role as chromosome custodians with distinct functions which ensure the error-free resolution of DNA damage through HR repair (62). The biochemical mechanism by which BRCA2 stabilizes the structure of replication-associated DNA lesions controlling the activity and assembly of the essential recombinase RAD51 has been elucidated in detail (63). Yet the precise mechanism by which BRCA2 exerts its tumor suppressor function remains unclear.

Compelling evidence is emerging which shows additional functions of BRCA2 beyond HR-mediated DNA repair, including those involved in protection of telomere integrity and stalled replication forks, in controlling cell cycle progression, transcription and mitophagy. Of particular relevance for its tumor suppressor role are evidences showing that BRCA2 can promote RCD through a mechanism conserved from yeast to mammals and specifically anoikis in human epithelial cells (35). Supporting this evidence is the observation that, although loss of heterozygosity (LOH) in somatic cells is considered essential for tumorigenesis in cancers harboring dysfunctional BRCA1/2, germline heterozygous BRCA2 mutations appear to suffice for carcinogenesis in several tissues, with LOH either absent or occurring relatively late in cancer progression [(62) and refs therein].

Taking advantage of a yeast model, we investigated potential new BRCA2 functions, which might have a role in its oncosuppressive mechanism, carrying out a preliminary analysis of genes differentially expressed in cells expressing or non-expressing BRCA2 either during cell growth or death. We confirmed that BRCA2 function is mainly activated in response to both DNA replication and death-inducing stress. In fact, BRCA2 over-expression does not affect the transcriptional read-out during normal cell growth whereas a reconfiguration of the transcriptional profile is clearly observed after induction of AA-RCD in BRCA2-expressing cells with apparent differences as compared with cells without BRCA2 (53, 55).

Indeed, a number of diverse proteins have already been reported to physically interact with BRCA2, as reported in (62). These BRCA2-interacting proteins predominantly belong to the class of proteins involved in maintenance of genome stability. Our study performed on yeast cells dying in response to acetic acid unveiled an expanded protein network interacting with BRCA2 as inferred from up- and down-regulated genes in cells undergoing AA-RCD (**Figures S2, S3**, respectively). In this respect, it is of note that *en route* to cell death process, BRCA2 expression apparently down-regulates genes involved in cell cycle progression, DNA repair mechanisms and oxidative stress response (**Figure S3**) strongly suggesting that distinct cell stress responses are activated by BRCA2 depending on the kind of stress. This experimental finding supports the hypothesis that BRCA2 may contain one or more intrinsically disordered regions, which lack a defined three-dimensional structure in isolation but, instead, tend to acquire more stable conformations when they bind to other macromolecules (64). These proteins belong to a small subset of proteins that serve as dynamic “hubs” for multiple macromolecular complexes, which can act as segmental entities, in which distinct and sometimes intrinsically disordered regions enter into different physical interactions to perform distinct biological functions [(62) and refs therein].

**Figure 3:**
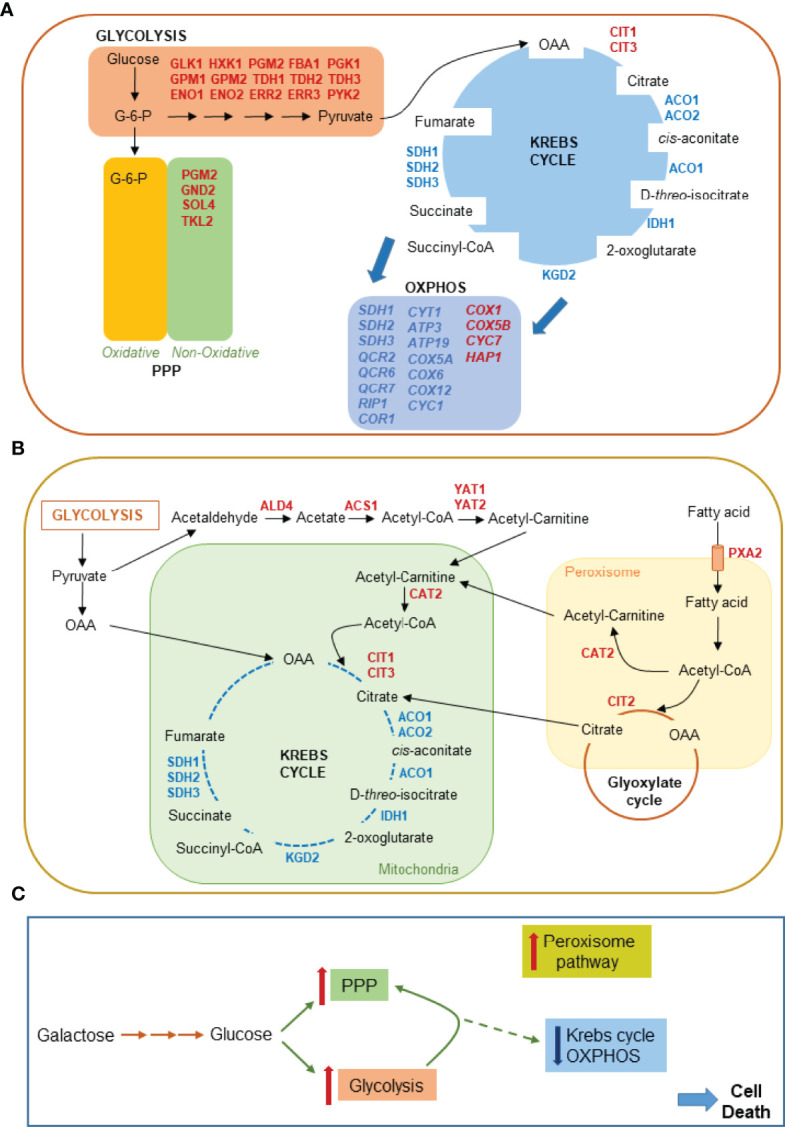
Metabolic pathways affected by BRCA2 expression in yeast cells undergoing AA-RCD as inferred from RNA-seq transcriptomic profiling. **(A)** 25 genes up-regulated in central carbon metabolism pathways, i.e., glycolysis, pentose phosphate pathway (PPP), Krebs cycle and oxidative phosphorylation (OXPHOS) are displayed in red. 23 genes down-regulated in Krebs cycle and OXPHOS are indicated in blue. **(B)** Mitochondrial RTG pathway target genes up-regulated in the cytosol, mitochondria and peroxisomes of AA-RCD cells expressing BRCA2 are displayed in red. Also included in the display in blue 8 genes downregulated in Krebs cycle as in **(A)**. **(C)** Krebs cycle and OXPHOS flux from glucose is strongly repressed (broken line) whereas glycolytic and PPP flux increases in yeast cells expressing BRCA2 *en route* to death.

In case of yeast cell death induced by acetic acid, which is a *bona fide* model of mammalian mitochondrial apoptotic pathway, the effect of BRCA2 expression apparently causes a metabolic reconfiguration characterized by increase in glycolytic flux, suppression of mitochondrial function and ensuing activation of retrograde response following mitochondrial dysfunction (**Figure 3**). Two genes, *COX5b* and *CYC7*, which are typically de-repressed under hypoxic conditions, were found up-regulated. Most likely this occurred *via* a mechanism distinct from hypoxic signaling, in response to oxidative stress conditions (65).

Whereas down regulation of cell cycle, DNA repair and oxidative stress response genes is in agreement with increased AA-RCD observed in BRCA2-expressing yeast cells [(35) and **Figure S1**], mitochondrial dysfunction and ensuing mitochondrial RTG pathway activation deserve specific discussion. The RTG pathway is commonly activated in response to various physiological stresses including glutamate depletion and loss of respiration capacity [for refs see (60)] as well as regulated cell death induction (66–68) and ER stress (69). RTG pathway activation upregulates the transcription of several genes coding for enzymes catalyzing anaplerotic reactions of the Krebs cycle to replenish alpha-ketoglutarate (αKG) pool and other Krebs cycle intermediates through cytoplasmic acetyl-CoA production (60, 70), as revealed here by RNA-seq analysis (**Figure 3B**). Krebs cycle intermediates may play important roles including those relevant to biosynthetic purposes as well as functions controlling chromatin modifications, DNA methylation, the hypoxic response, and immunity in higher eukaryotes (71). Thus, RTG pathway activation can provide the cell with an adaptive response to deadly signals promoting cell survival. However, yeast evades cell death in response to acetic acid or endoplasmic reticulum stress due to concomitant RTG pathway activation and mitochondrial respiration de-repression (66, 68, 69). Thus, the repression of genes involved in respiratory metabolism due to BRCA2 expression in yeast cells *en route* to death (**Figures 3C**, **S2**) could explain the apparent inability of RTG pathway activation to rescue yeast from cell death. Since the transcriptome profile observed in yeast cells undergoing AA-RCD in the presence of BRCA2 is indicative of cell cycle arrest (**Figure S3**) it is also of note that mitochondrial RTG signaling can inhibit the survival during prolong S/G2 arrest in yeast (72). Whether the role of RTG pathway in the checkpoint regulation is conserved in higher organisms needs further investigation. Nevertheless, it could be assumed that tightening of cell cycle arrest by activated RTG pathway under stress condition can inhibit growth of cells with dysfunctional mitochondria resulting in a loss of colony-forming ability observed in this study (**Figure S1**). Thus, yeast can be used to study structural and functional features of BRCA2 in cell death regulation. Future investigation will clarify whether BRCA2-dependent central carbon metabolism reprogramming is relevant to its tumor suppression function.

## Data Availability Statement

The datasets presented in this study can be found in online repositories. The names of the repository/repositories and accession number(s) can be found in the article/**Supplementary Materia**l.

## Author Contributions

SG, GP, AD, and EP contributed to conception and design of the study, AC, NG, and CM performed experiments and RNA sequencing, AL, CL, AB, and EP analyzed trasncriptomic data and organized the database. SG and AC wrote the first draft of the manuscript. AD, EP, AL, and CL wrote sections of the manuscript. All authors contributed to manuscript revision, read, and approved the submitted version.

## Funding

The work has been supported by ELIXIR infrastructure and MUR FOE grant “Sviluppo di protocolli innovativi e applicazione di nuovi strumenti -omici nei pazienti orfani di diagnosi” to CNR.

## Conflict of Interest

The authors declare that the research was conducted in the absence of any commercial or financial relationships that could be construed as a potential conflict of interest.

## Publisher’s Note

All claims expressed in this article are solely those of the authors and do not necessarily represent those of their affiliated organizations, or those of the publisher, the editors and the reviewers. Any product that may be evaluated in this article, or claim that may be made by its manufacturer, is not guaranteed or endorsed by the publisher.
