# Supplementary material for: Yeast as a Model to Unravel New BRCA2 Functions in Cell Metabolism
Source: Front Oncol. 2022 Jun 6;12:908442. doi: 10.3389/fonc.2022.908442 (PMC9207209; doi:10.3389/fonc.2022.908442)
Supplement: Supplementary file 1 [file DataSheet_1.docx]

**SUPPLEMENTARY MATERIALS**

**Strain, culture condition and acetic acid treatment**

The *S. cerevisiae* strain used in this work was W303-1B (MATα, *ade2 leu2 his3 trp1ura3*). Cells were transformed by lithium acetate procedure with the episomal plasmid pYES2 containing or not the human BRCA2 cDNA under the control of yeast galactose-inducible promoter pGAL1 (Guaragnella et al 2014). The transformants, named Y-pYES and Y-BRCA2 respectively, were selected on synthetic complete medium containing 2% dextrose without uracil (SCD-URA). For maintenance of this strain, the growth is possible on YPD (1 % yeast extract, 2 % bactopeptone and 2% dextrose) or SCD-URA.

For improved BRCA2 expression we used the experimental conditions described in [1] modified as follows. In brief, a single colony of Y-pYES and Y-BRCA2 was grown in SC containing 3% glycerol, 0.5% ethanol and 0.5% dextrose without uracil (SC-GED-URA) for about 16 hours at 30° at 160 rpm. Cells were washed twice to remove medium and re-suspended in SC containing 2% galactose (SC-Gal) at initial OD_600_=0.5 for induction and incubated at 26° C at 160 rpm for about 16 hours.

For induction of RCD with acetic acid (AA) treatment, a single colony of Y-pYES and Y-BRCA2 was grown in SC-GED-URA for about 16 hours at 30 °C at 160 rpm. Cells were washed twice to remove medium and re-suspended in SC-Gal from initial OD_600_=0.5. Then, cells were resuspended (10^7^ cells/ml) in SC-Gal adjusted to pH 3.00 with HCl, in the absence (control) or in the presence of AA and incubated for different times at 26° C [2]. Cell viability was determined by measuring colony forming units (cfu) after two days of growth on YPD plate at 30 °C. In this experimental set-up yeast cells were grown in conditions different from those used in [3]. Thus, we tested different concentrations of AA (150, 200, 300, 400 mM) to find the AA concentration at which the loss of viability of yeast cells expressing BRCA2 was significantly higher than that of cells without BRCA2. We chose 300 mM acetic acid to induce yeast cell death based on the cell viability data shown in figure S1 that mirrors the results obtained in [3].

**RNA extraction**

Total RNA was extracted from 1x10^8^ yeast cells treated for 1 hours at 30° with 200U of zymolyase in Sorbitol Buffer using the Rneasy Mini/Plus extraction kit (Qiagen), according to the manufacturer’s instructions. RNA was quantitatively and qualitatively evaluated using NanoDrop 2000c (Thermo Fisher Scientific) and Agilent Bioanalyzer 2100 (Agilent Tecnologies), respectively.

**RNA sequencing**

Directional polyA+ RNA libraries were prepared from 1 µg of total RNA using the SureSelect Strand-Specific RNA Library Prep for Illumina Multiplexed Sequencing (Agilent Technologies) according to the manufacturer’s protocol. Libraries were qualitatively checked on the Agilent 2100 platform and quantified on the Nano-Drop 3300 Fluorospectrometer (Thermo-Scientific) using the Quant-IT PicoGreen assay kit (Life-Technologies). Equimolar quantities of cDNA libraries were pooled and subjected to 2 × 120 bp paired-end sequencing on the NextSeq 500 platform (Illumina), generating on average 18 million of reads per sample.

### Preprocessing and analysis of RNA-seq data

At least 4 replicates for each experimental condition were analyzed. Raw data were initially quality-checked using FastQC and trimmed by cutadapt in order to remove low quality bases and adapters. High quality reads were aligned onto the yeast genome (assembly R64 from strain S288C) using the ultrafast STAR program. Raw counts for known genes were performed by FeatureCounts while differential expression analysis was carried out using DESeq2. Differentially expressed genes were selected if log2-fold change | ≥ 1.5 and p-value<0.05)|. Yeast gene annotation was performed by means of the blast2go module embedded with OmicsBox and according to the following steps: tBLASTx to find homologous sequences; Gene Ontology mapping to retrieve GO terms; Annotation Prediction to select reliable functions. Gene Ontology (GO) enrichment was carried out by means of the PANTHER Classification System (www.pantherdb.org). Biological pathway analysis was performed using the Kyoto Encyclopedia of Genes and Genomes (KEGG, http:// www.kegg.jp/) database.

**Data availability**

Raw reads used in this study are publicly available through the SRA BioProject PRJNA818194.

**References**

1. Liu, J.; Doty, T.; Gibson, B.; Heyer, W.-D. Human BRCA2 Protein Promotes RAD51 Filament Formation on RPA-Covered Single-Stranded DNA. *Nat Struct Mol Biol* **2010**, *17*, 1260–1262, doi:10.1038/nsmb.1904.

2. Giannattasio, S.; Guaragnella, N.; Corte-Real, M.; Passarella, S.; Marra, E. Acid Stress Adaptation Protects Saccharomyces Cerevisiae from Acetic Acid-Induced Programmed Cell Death. *Gene* **2005**, *354*, 93–98, doi:10.1016/j.gene.2005.03.030.

3. Guaragnella, N.; Marra, E.; Galli, A.; Moro, L.; Giannattasio, S. Silencing of BRCA2 Decreases Anoikis and Its Heterologous Expression Sensitizes Yeast Cells to Acetic Acid-Induced Programmed Cell Death. *Apoptosis* **2014**, *19*, 1330–1341, doi:10.1007/s10495-014-1006-z.

**Supplementary figure captions.**

**Figure S1.** Cell viability of Y-pYES and Y-BRCA2 cells treated with 300 mM AA**.**

**Figure S2.** Interaction network of up-regulated genes (score > 0.7).

**Figure S3.** Interaction network of down-regulated genes (score > 0.7).

**Table S1. (Data sheet – Excel file)** List of significant Gene Ontology terms deriving from the DEGs analysis.

**Table S2.** List of up- and down-regulated genes involved in the functional classification.

| **PATHWAY** | **No. of DEGs** | **Up-regulated genes** | **Down-regulated genes** |
| --- | --- | --- | --- |
| Metabolic pathways | 202 | COX1, ACS1, GDH3, PHO11, PRX1, UGA2, ETR1, CHS2, PHO5, TKL2, TPS1, LEU2, GLK1, CIT2, PGK1, GPM2, DIA3, THI3, LYS20, GUD1, THI13, TPI1, TPS2, AMD2, DPL1, IPK1, GLC3, DLD3, FAA2, GPP2, DAK2, GSY1, HXK1, PMA1, PDE1, ADH4, NQM1, PDC6, CTT1, THI4, TDH3, SOL4, ENO1, GND2, INM1, GRE3, ENO2, FAA3, CAB2, SGA1, COX5B, AYR1, SUC2, TDH1, LSB6, GSH1, INO1, TDH2, UTR1, OPI3, MET5, PGU1, THI11, SOR1, UGP1, OAR1, FBA1, RMA1, GPM1, GLG1, GPT2, MHT1, PUT1, GSY2, MET17, CAR2, HMG2, DAK1, TSL1, ADI1, FMS1, PGM2, ALD3, ALD2, GAD1, TPS3, URA10, ERR3, IDP3, MET2, BIO4, BIO3, MAN2, THI20, GCY1, HIS3, CPA1, PYK2, ALD4, HSP33, HST2, PMA2, HSP32, ERR2, CIT3, ATH1, pyridoxine-4-dehydrogenase, GPH1, GDB1 | CYS3, PMT2, URA7, COR1, PRS4, MNN2, CHS3, CDS1, ATP3, MIS1, TYR1, ALG7, ARO4, DUT1, TSC10, APA1, CHA1, ELO2, THR4, PSA1, PMT5, QRI1, FAP7, DLD2, KGD2, QCR7, RIP1, PMI40, SAH1, RNR1, ADK2, SEC53, QCR6, ERG4, OCH1, ARO2, HXK2, ERG25, ASN2, ERG11, COX6, FUR1, ARO9, DCD1, BAT1, IMD2, HIS6, RNR2, RPE1, MNN11, ILV3, HAM1, PMT4, SDH3, SDH1, URA1, PCK1, SDH2, MEU1, COX12, ERG3, ACS2, ACO1, ELO3, IMD3, ERG6, OST6, APT1, IMD4, ERG5, ERG2, GUA1, ADH6, NCE103, IDH1, COX5A, NRK1, AAH1, ATP19, CYT1, CDC21, LEU9, IDH2, LIP5, MNN9, KTR6, BTS1, CAR1, ALG5, SAM4, ARO7, DPM1, QCR2 |
| Ribosome | 137 | - | RPS9B, RPL21A, MRPL32, RPS14A, RPL31A, RPP1A, RPL13A, RPP1B, RPL41A, RPL41A, RPL35A, RPL35A, MRPL11, RPS11A, RPS11A, RPS13, MRPL1, MRPL7, RPP2B, MRP20, RPS17B, RPL27B, RPL37B, RPL12A, RPL12A, RPL34A, RPS8B, RPS8B, RPL23B, RPL23B, RPS26B, RPL29, RPL24A, MNP1, RPL7A, RPL28, RPS2, RPL9A, RPS26A, RPS25A, RPL26B, RPL11B, RPL24B, RPS0A, MRPL9, RPL14B, RPS20, RPL8A, RPL27A, RPS27B, RPL2B, RPL2B, RPL34B, RPS24B, RPS24B, RPL16A, MRPL8, MRPL49, RPS21B, RPL17B, RPL39, RPS22A, RPS5, RPS4A, RPS4A, RPL14A, RPS27A, RPL17A, RPS21A, RPL40B, RPL40B, RPL8B, RLP24, RPL15A, RPS0B, RPL22A, RPL10, RPS31, RPL37A, RPL38, RPS25B, RPP0, RPL26A, RPL31B, RPS1A, RPL6B, MRPL39, RPS17A, RPS18B, RPS18B, RPS1B, RPL6A, RPL13B, RPS16A, RPS16A, MRPS17, MRPL24, RPL36A, RPS10B, RLP7, RPL9B, RPL16B, RPS7B, RPL42A, RPL42A, RPS3, MRPL19, RPS19B, RPP2A, RPS15, RPL18A, RPS19A, RPL25, RPL3, RPS7A, RPS28A, RPS30B, RPL33B, RPS10A, RPL20B, RPL20B, RPS12, RPL21B, RPS9A, RPS6A, RPS6A, RPL5, RPL33A, MRPL40, RPL1A, RPL1A, RPL36B, RPL43A, RPL43A, RPL11A, RPS23B, RPS23B |
| Biosynthesis of secondary metabolites | 107 | ACS1, TKL2, TPS1, LEU2, GLK1, CIT2, PGK1, GPM2, THI3, LYS20, TPI1, TPS2, GLC3, GSY1, HXK1, ADH4, NQM1, PDC6, CTT1, TDH3, SOL4, ENO1, GND2, INM1, ENO2, SUC2, TDH1, INO1, TDH2, OPI3, UGP1, OAR1, FBA1, GPM1, GLG1, GPT2, MHT1, PUT1, GSY2, MET17, CAR2, HMG2, TSL1, PGM2, ALD3, ALD2, GAD1, TPS3, ERR3, IDP3, MET2, HIS3, PYK2, ALD4, ERR2, CIT3, ATH1, GPH1, GDB1 | CYS3, PRS4, CDS1, TYR1, ARO4, CHA1, ELO2, THR4, PSA1, FAP7, KGD2, LPP1, PMI40, ADK2, SEC53, ERG4, ARO2, HXK2, ERG25, ASN2, ERG11, ARO9, BAT1, IMD2, HIS6, RPE1, ILV3, SDH3, SDH1, PCK1, SDH2, ERG3, ACS2, ACO1, ELO3, IMD3, ERG6, IMD4, ERG5, ERG2, ADH6, IDH1, LEU9, IDH2, BTS1, CAR1, SAM4, ARO7 |
| Carbon metabolism | 40 | ACS1, TKL2, GLK1, CIT2, PGK1, GPM2, TPI1, DAK2, HXK1, NQM1, CTT1, TDH3, SOL4, ENO1, GND2, ENO2, TDH1, TDH2, FBA1, GPM1, MET17, DAK1, ERR3, IDP3, PYK2, ERR2, CIT3 | PRS4, CHA1, KGD2, HXK2, RPE1, SDH3, SDH1, PCK1, SDH2, ACS2, ACO1, IDH1, IDH2 |
| Biosynthesis of amino acids | 41 | TKL2, LEU2, CIT2, PGK1, GPM2, LYS20, TPI1, NQM1, TDH3, ENO1, ENO2, TDH1, TDH2, FBA1, GPM1, MET17, ERR3, IDP3, MET2, HIS3, PYK2, ERR2, CIT3 | CYS3, PRS4, TYR1, ARO4, CHA1, THR4, ARO2, ASN2, BAT1, HIS6, RPE1, ILV3, ACO1, IDH1, LEU9, IDH2, CAR1, ARO7 |
| Cell cycle | 41 | CLN3, PHO11, PHO5, PCL9, CDC20, CLB1, MET30, PHO80 | LTE1, BRN1, CYC8, KCC4, MRC1, TUP1, MCD1, CDC7, DUN1, PCL2, DBF4, PDS1, YHP1, GIN4, SMC1, CDC14, CDC26, CLB6, IRR1, SMC3, SWE1, BFA1, HSL1, CDC45, YCS4, YOX1, CLN1, PCL1, BUB3, SLK19, RAD53, CLN2, CLB5 |
| MAPK signaling pathway | 29 | PKH1, STE2, MTL1, ROM1, CTT1, CLB1, SDP1, MSN4, TUS1, SST2, PKH2, WSC3 | CYC8, PAF1, TUP1, MKC7, DIG2, SHO1, MSB2, CLB6, SWE1, HSL1, SPA2, CDC42, CLN1, WSC2, CLA4, CLN2, CLB5 |
| Glycolysis / Gluconeogenesis | 27 | ACS1, GLK1, PGK1, GPM2, THI3, TPI1, HXK1, ADH4, PDC6, TDH3, ENO1, ENO2, TDH1, TDH2, FBA1, GPM1, PGM2, ALD3, ALD2, ERR3, PYK2, ALD4, ERR2 | HXK2, PCK1, ACS2, ADH6 |
| Oxidative phosphorylation | 18 | COX1, PMA1, COX5B, PMA2, CYC1 | COR1, ATP3, QCR7, RIP1, QCR6, COX6, SDH3, SDH1, SDH2, COX12, COX5A, ATP19, CYT1, QCR2, CYC7 |
| Starch and sucrose metabolism | 18 | TPS1, GLK1, TPS2, GLC3, GSY1, HXK1, SGA1, SUC2, UGP1, GLG1, GSY2, TSL1, PGM2, TPS3, ATH1, GPH1, GDB1 | HXK2 |
| DNA replication | 17 | - | RFA1, POL12, RFC5, POL30, POL3, CDC9, PRI1, POL31, POL32, PRI2, RAD27, RNH201, POL1, RFC3, RFA2, RFC4, DPB2 |
| Purine metabolism | 17 | GUD1, PDE1, PGM2, PYK2 | PRS4, APA1, FAP7, RNR1, ADK2, IMD2, RNR2, HAM1, IMD3, APT1, IMD4, GUA1, AAH1 |
| Pyruvate metabolism | 13 | ACS1, LYS20, DLD3, ALD3, ALD2, PYK2, ALD4, HSP33, HSP32 | DLD2, PCK1, ACS2, LEU9 |
| Mismatch repair | 13 | - | RFA1, RFC5, POL30, POL3, CDC9, MSH6, POL31, POL32, PMS1, RFC3, RFA2, MSH2, RFC4 |
| Citrate cycle (TCA cycle) | 11 | CIT2, IDP3, CIT3 | KGD2, SDH3, SDH1, PCK1, SDH2, ACO1, IDH1, IDH2 |
| Base excision repair | 9 | - | POL30, POL3, CDC9, POL31, POL32, RAD27, UNG1, OGG1, DPB2 |
| Homologous recombination | 8 | RAD50 | RFA1, RDH54, POL3, RAD51, POL31, POL32, RFA2 |
| Nucleotide excision repair | 12 | RAD28 | RFA1, RFC5, POL30, POL3, CDC9, POL31, POL32, RFC3, RFA2, RFC4, DPB2 |
| Pentose phosphate pathway | 8 | TKL2, NQM1, SOL4, GND2, FBA1, PGM2 | PRS4, RPE1 |
| Peroxisome | 11 | YAT1, FAA2, YAT2, CTT1, FAA3, PEX2, PXA2, SYM1, CAT2, IDP3 | RSM26 |
| Pyrimidine metabolism | 10 | URA10, CPA1 | URA7, DUT1, RNR1, FUR1, DCD1, RNR2, URA1, CDC21 |
| RNA degadation | 10 | ENO1, ENO2, DCS1, ERR3, ERR2 | PAT1, MTR3, MTR4, TRF5, DCP1 |
