# Supplementary figures and images for: Yeast as a Model to Unravel New BRCA2 Functions in Cell Metabolism
Source: Front Oncol. 2022 Jun 6;12:908442. doi: 10.3389/fonc.2022.908442 (PMC9207209; doi:10.3389/fonc.2022.908442)

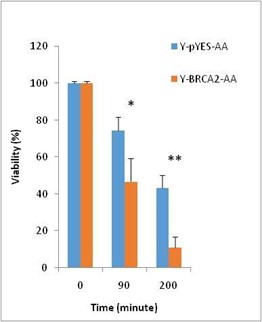

Supplement: Supplementary file 2 [file Image_1.jpeg]

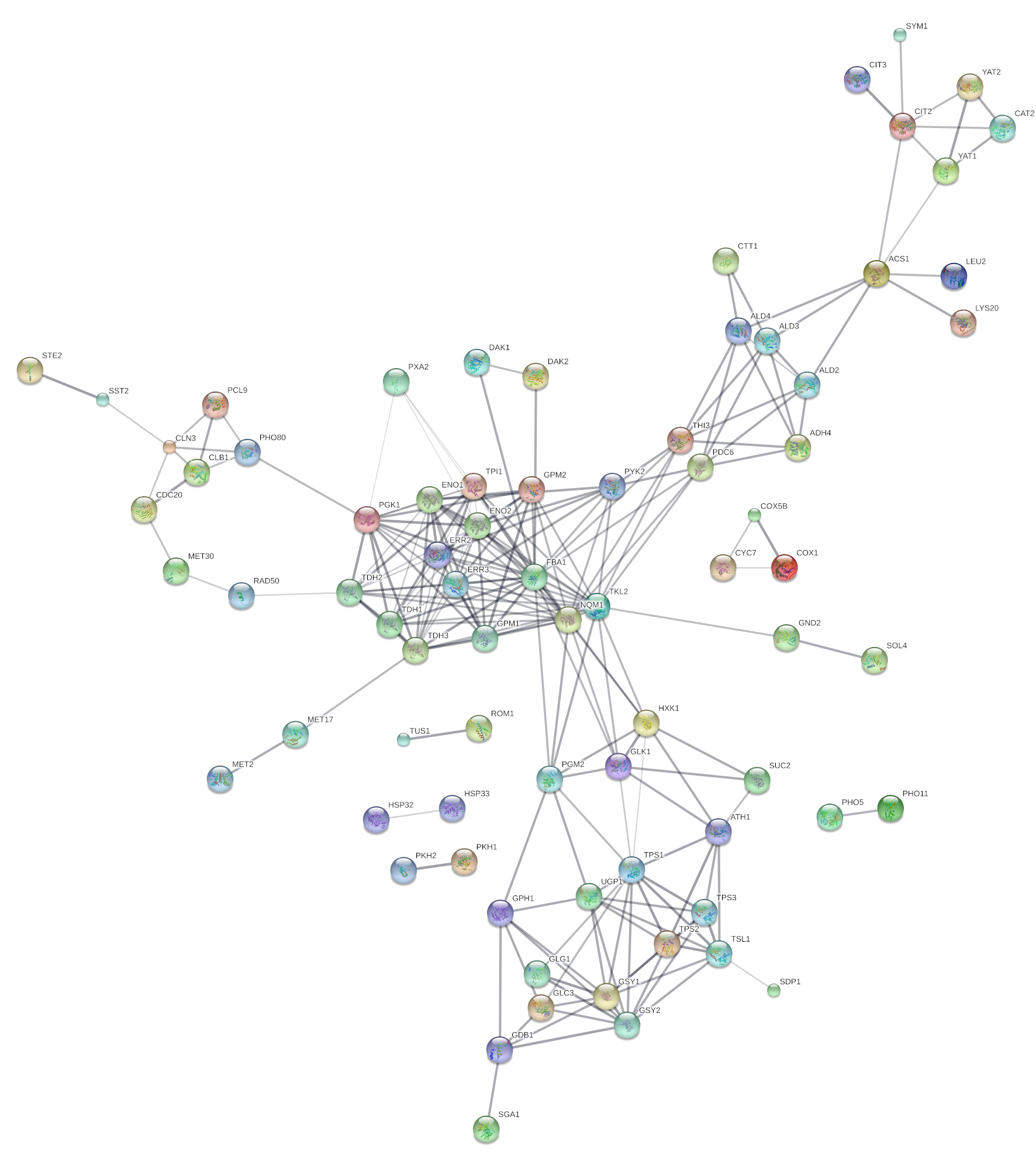

Supplement: Supplementary file 3 [file Image_2.tif]

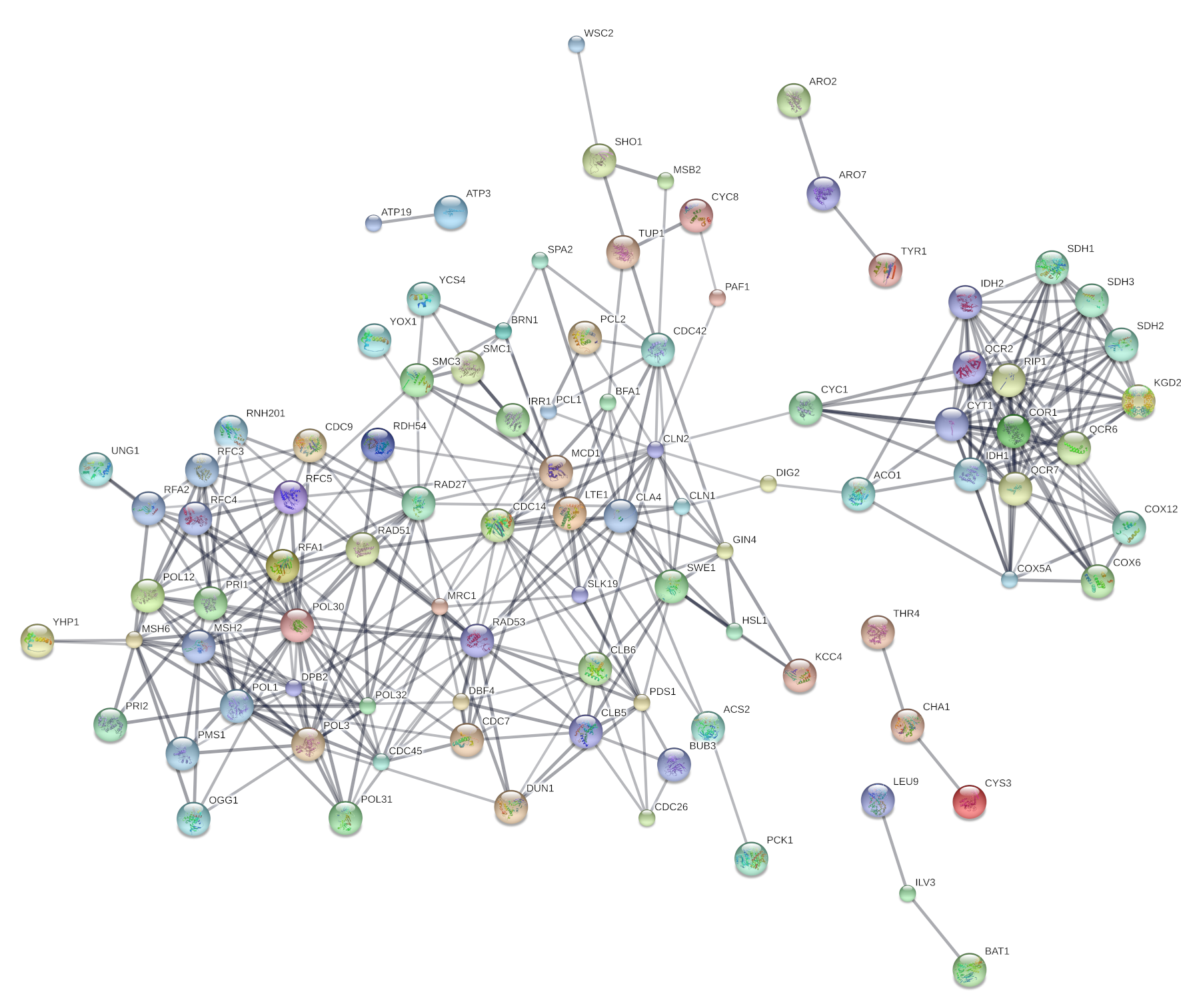

Supplement: Supplementary file 4 [file Image_3.tif]
